# Microbial communities associated with ferromanganese nodules and the surrounding sediments

**DOI:** 10.3389/fmicb.2013.00161

**Published:** 2013-06-25

**Authors:** Benjamin J. Tully, John F. Heidelberg

**Affiliations:** Department of Biological Science, Marine and Environmental Biology, Dornsife College of Letters, Arts, and Sciences, University of Southern CaliforniaLos Angeles, CA, USA

**Keywords:** ferromanganese nodules, polymetallic nodules, microbial ecology, 16S rRNA, community analysis

## Abstract

The formation and maintenance of deep-sea ferromanganese/polymetallic nodules still remains a mystery 140 years after their discovery. The wealth of rare metals concentrated in these nodules has spurred global interest in exploring the mining potential of these resources. The prevailing theory of abiotic formation has been called into question and the role of microbial metabolisms in nodule development is now an area of active research. To understand the community structure of microbes associated with nodules and their surrounding sediment, we performed targeted sequencing of the V4 hypervariable region of the 16S rRNA gene from three nodules collected from the central South Pacific. Results have shown that the microbial communities of the nodules are significantly distinct from the communities in the surrounding sediments, and that the interiors of the nodules harbor communities different from the exterior. This suggests not only differences in potential metabolisms between the nodule and sediment communities, but also differences in the dominant metabolisms of interior and exterior communities. We identified several operational taxonomic units (OTUs) unique to both the nodule and sediment environments. The identified OTUs were assigned putative taxonomic identifications, including two OTUs only found associated with the nodules, which were assigned to the *α-Proteobacteria*. Finally, we explored the diversity of the most assigned taxonomic group, the *Thaumarchaea* MG-1, which revealed novel OTUs compared to previous research from the region and suggests a potential role as a source of fixed carbon for ammonia oxidizing archaea in the environment.

## Introduction

Ferromanganese/polymetallic nodules form at the sediment-water column interface in deep-sea environments (4–6000 m). Generally small in size (1–5 cm) and formed as concentric laminated structures, they are primarily composed of manganese (Mn), iron (Fe), and a large number of other metals, including copper, nickel, zinc, and titanium; however, composition varies by nodule and oceanic province. Despite their small size, the global estimate for metal content in ferromanganese (FeMn) nodules is 2 × 10^14^ kg each of Fe and Mn (Somayajulu, 2000). Recently, an increase in the value of rare earth metals has stimulated an interest in mining these resources. The removal of FeMn nodules from the seafloor could have unknown ramifications on the environment, since the processes governing nodule formation and maintenance and the role nodules play in supporting the adjacent biosphere is poorly understood.

The formation process for FeMn nodules has been a scientific unknown since their discovery in the 1870's (Murray, 1891). Emphasis had been placed on abiotic processes, with formation times on the order of a few mm ·10^6^ yr^−1^ based on radiometric data (Kerr, 1984). But new quantification techniques have reduced the estimates of formation time to a few mm ·10^3^ yr^−1^ (Boltenkov, 2012). As our understanding of the role microorganisms play in geochemical processes has increased, research has started to shift toward determining if biotic processes play a role in nodule formation. Much of the recent evidence of a microbial component to nodule geochemistry revolves around visual inspection of nodules using scanning electron microscopy. These studies have identified different exolithic and endolithic morphotypes of microorganisms (Zhang et al., 2002; Lysyuk, 2008; Wang et al., 2009a,b). Like most deep-sea environments, little is known about the physiologies and metabolisms of microorganisms associated with FeMn nodules or the impact these microbial processes may have on global ocean metal chemistry.

Previous work on FeMn nodules comes from samples collected from the Clarion-Clipperton Zone in the eastern North Pacific (Wang et al., 2012). An equally large FeMn nodule province exists within the central South Pacific Gyre (SPG), where FeMn nodules can occupy as much as 70% of the exposed surface sediment. The SPG has the lowest primary productivity and sedimentation rates of the major ocean gyres and, as a direct result, has extremely oligotrophic, recalcitrant underlying sediment (D'Hondt et al., 2009). FeMn nodules are responsible for a number of abiotic processes, including the degradation refractory organic compounds in to labile, low molecular weight organic compounds (Sunda and Kieber, 1994). These metabolically available compounds may then stimulate microbial respiration. Nodules also act as concentrators of metallic dications (Ni^2+^, Cu^2+^, Zn^2+^) (Dick et al., 2008) and anionic forms of phosphorous, vanadium, molybdenum, and tungsten (Koschinsky and Halbach, 1995), many of which are important co-factors for key biochemical processes. Thus, FeMn nodules may play an important role in the degradation of buried organic compounds and the global carbon cycle.

Previous attempts to isolate genetic material from marine FeMn nodules have been unsuccessful, with the exception of a single published 16S rRNA gene sequence (Wang et al., 2009c). We sampled nodules and sediments from three different sites in the SPG to gain a greater understanding of the biogenic controls associated with nodule formation and cycling. The V4 hypervariable region of the 16S rRNA gene was amplified from DNA extracted from different layers within each nodule and the surrounding sediment and sequenced using 454 sequencing. Deep sequencing the microbial community allowed us to determine the dominant 16S rRNA gene sequences and compare how the community structure varied between nodules from different sites and by the source layer from within the nodules. We found that the most abundant group of organisms could be assigned to the *Thaumarchaea*, but that this group was extremely diverse in the nodule/sediment ecosystem. We found that the microbial communities associated with the nodule were distinct from the communities present in the sediment and the nodule communities varied based on sampling site. Further, the microbial communities were significantly different between nodule layers.

## Materials and methods

### Sample collection

Sediment and FeMn nodules were collected as part of Expedition Knox-02RR (December 2006–January 2007 aboard the R/V *Roger Revelle*). Nodules and surface sediments were collected from SPG2, 9, and 10, with an additional sediment sample collected from SPG3 (Table 1). The largest nodule collected measured 6.5 cm in diameter. Nodules and corresponding sediments were aseptically sampled from a multicore on the catwalk, as samples were brought onboard. Sediments from 0 to 5 cm were sampled from the same cores from which the nodules were obtained and stored at −80°C. Nodules were rinsed gently with 0.2 μm filtered and autoclaved ambient bottom water to remove sediment adhering to the surface. A flame-sterilized hammer and chisel were used to aseptically section the nodules based on visual changes in strata (delimited as either outer layer, inner layer, or core, where applicable). Further subsamples were generated and stored in 1.5 mL cyrovials and stored at −80°C.

**Table 1:** **Sampling site locations and depths**.

**Site**	**Location**	**Meters below sea level**
SPG2	26° 03.0896′ S, 156° 53.6472′ W	5127
SPG3	27° 56.539′ S, 148° 35.388′ W	4852
SPG9	38° 03.6904′ S, 133° 05.5072′ W	5697
SPG10	39° 18.617′ S, 139° 48.036′ W	5283

### DNA extraction

Extraction of DNA from nodules proceeded using a modified phenol-chloroform extraction method (Zhou et al., 1996; Juretschko et al., 1998). Approximately 0.5 mL of sample was resuspended in 675 μL of 2% CTAB (cetyltrimethylammonium bromide) lysis buffer [100 mM Tris, 100 mM EDTA, 250 mM Na_2_PO_4_, 1.5 M NaCl, brought to 40 mL and adjusted to pH 8.0; addition of 2% CTAB; diluted to 50 mL total volume and autoclaved] and vortexed thoroughly for 30 s. To each slurry, 20 μL of proteinase K (800 units·mL^−1^) was added and incubated horizontally for 30 min at 50°C. To each sample, 150 μL of 10% SDS was added and incubated further for 120 min at 65°C followed by the addition of 600 μL of PCI (phenol:chloroform:isoamyl alcohol) 25:24:1 and incubation for 20 min at 65°C. Samples where then centrifuged for 10 min at 10,000 × g. The upper layer was transferred to a new tube (care was taken to avoid transferring material from the interface or below), 0.7 volumes of isopropanol was added, and incubated for 60 min at room temperature. Samples were again centrifuged at 10,000 × g for 15 min, 0.5 mL of cold 70% ethanol was added, and then centrifuged for an additional 5 min. Following removal of the supernatant, the pellet was left to air dry in a fume hood for 15–30 min (as necessary) and resuspended in 30 μL of sterile, DNase-free H_2_O. Samples were then quantified (3 μL) using the Qubit 1.0 fluorometer and the Qubit dsDNA HS Assay Kit (Life Technologies).

Due to low yield using the described phenol-chloroform method, extraction of DNA from sediment samples was performed using the MoBio PowerLyzer PowerSoil DNA kit following the manufacturer's protocol, and quantified as above.

All samples with >0.1 ng/μL final DNA concentration were cleaned and concentrated using the ZYMO Clean + Concentrator 5 (6:1 DNA Binding Buffer, as per suggested protocol) and samples were resuspended in 20 μL of sterile, DNase-free H_2_O.

### Sample amplification and pyrosequencing

Samples were amplified using PCR, targeting the V4 region of the 16S rRNA gene. All amplifications were performed using the FastStart High Fidelity PCR System (Roche). Initial amplification was performed in triplicate using a forward fusion primer, [5′-**CGTATCGCCTCCCTCGCGCCATCAG**xxxxxxxxxx(x)GTGYCAGCMGCCGCGGTA-3′] designed to incorporate the Roche 454 Titanium Adapter A sequence (bold), a multiplexing identifier (MID) sequence, the U515 forward primer (underlined) (Wang and Qian, 2009), and a U1048 reverse primer (5′-CGRCRCCATGYANCWC-3′) modified from Huber et al. (2007). Each 50 μL PCR reaction was composed of: forward primer (0.8 μM), reverse primer (0.8 μM), 0.2 mM dNTPs, 1X buffer #2, 15 μg of BSA (bovine serum albumin), PVP (polyvinylpyrrolidone, 0.5%), and 2.5 U Taq, with H_2_O up to 50 μL. If the template DNA had been cleaned and concentrated, 7 μL·reacion^−1^ was added, otherwise 10 μL·reaction^−1^ template DNA was added. Amplification proceeded on a thermocycler with the following heating steps: 95°C 4 min, 35 cycles of 95°C 30 s, 56°C 30 s, 72°C 1 min, 72°C 5 min, hold at 4°C.

Initial PCR products were pooled and the PCR product (~550 bp) was gel excised using the Qiagen Gel Extraction Kit (Qiagen) following the manufacturer's protocol. Excised DNA products were amplified in duplicate to generate sufficient material for pyrosequencing. The same forward primer was used, but the reverse primer (U1048R) had the 454 Roche Titanium Adapter B sequence (5′-**CTATGCGCCTTGCCAGCCCGCTCAG**-3′) added to the 5′ end. The second round of PCR amplification proceeded as above, with the following exceptions: [1] each primer was added at 0.6 μM final concentration; [2] no BSA or PVP was used; and, [3] 5 ng of template of DNA was added. The same settings were used for the thermocycler, except that amplification was only performed for 30 cycles.

PCR products were pooled and cleaned using the AMPure Bead XP (Agencourt) kit, following the manufacturer's protocol. Samples were quantified using PicoGreen and visualized using Agilent Bioanalyzer using the High Sensitivity (Agilent) chip. The various 16S rRNA gene amplicons were pooled following the recommend procedure in the Amplicon Library Preparation Method Manual (Roche, GS FLX Titanium Series, October 2009). Pyrosequencing was performed by EnGenCore (University of South Carolina, Columbia, SC) utilizing Titanium FLX chemistry. The raw data files have been deposited in the NCBI Sequence Read Archive with the accession number SRA082599.

Four of the samples with the same starting DNA were processed separately during the Titanium amplification and sequencing steps to provide technical replicates as to how well the procedure reproduced results for identical samples. While the absolute value of the number of sequences generated was different for each replicate, the relative abundance of the OTUs remained virtually the same (Supplemental Figure 1).

### Data analysis

Trimming, cleaning, and clustering of the 16S rRNA gene amplicon sequences generated via pyrosequencing was performed using mothur (V1.28) following the Schloss laboratory's standard operating procedure (SOP) (available at www.mothur.org) (Schloss et al., 2009, 2011; Schloss and Westcott, 2011). In brief, a combination of the programs trim.flows (set to 350 flows for FLX data), shhh.flows, and trim.seqs was used to identify high quality sequences and trimmed of any remaining adapter and primer sequences, using the recommended settings (allowing for 1 difference in the barcode, 2 differences in primer sequence, a maximum homopolymer length of 8 nucleotides, and a minimum length of 200 bp). Sequences remaining after these initial steps were aligned to a reference file generated using previously aligned SILVA 16S and 18S rRNA gene sequences (V111) from *Bacteria*, *Archaea* and *Eukarya*. The program screen.seqs was used to restrict the area of the sequences analyzed to an area immediately surrounding and including the V4 hypervariable region (13,861–23,959 bp of the aligned sequence). Following a series of steps to collapse related sequences in to more manageable numbers, groups were removed that did not have at least 1000 sequences remaining. The groups with >1000 sequences were processed using UCHIME to detect putative chimeric sequences by comparing all sequences to the most abundant sequences in the dataset (Edgar et al., 2011). Putative taxonomic assignments were derived using the classify.seqs program and filtered to remove sequences with the any taxonomic assignment to Chloroplast or Mitochondria. The programs dist.seqs and cluster (set to average neighbor) were used as described in the SOP.

The mothur tool was also used to analyze the processed sequences to determine α- and β-diversity measures and putative taxonomic assignment. For this downstream analysis, operational taxonomic unit (OTU) calls were made at the 99% identity level (“label = 0.01”), if applicable, all groups were randomly subsampled to 900 sequences (“size = 900”), and for any process were multiple iterations were required, 1000 iterations were used (“iters = 1000”). α-diversity was determined using the summary.single command, which estimated Good's coverage (Good, 1953) for the samples. Several calculators were used to determined β-diversity, including ThetaYC (θ_YC_), Jaccard dissimilarity, and Bray–Curtis dissimilarity. Statistical significance of these β-diversity measures were computed using parsimony, weighted Unifrac (Lozupone and Knight, 2005), and unweighted Unifrac (Lozupone and Knight, 2005). Non-metric multidimensional scaling (NMDS) ordination plots were constructed and statistical significance was determined using an analysis of molecular variance (AMOVA) test (Excoffier et al., 1992). OTUs were putatively classified using the classify.otu program.

### Phylogenetic tree construction

Representative sequences determined to putatively belong to the *Thaumarchaea* MG-1 group were generated in mothur. Representatives were aligned with CLUSTAL W (Thompson et al., 1994) to environmental sequences (Durbin and Teske, 2010) and sequenced members of the Phylum *Thaumarchaea* (Hallam and Konstantinidis, 2006; Hatzenpichler et al., 2008; Walker et al., 2010) and trimmed to a region (289 bp) that included the V4 hypervariable region of the 16S rRNA gene. Maximum likelihood trees were constructed with 1000 bootstraps using the Kimura (K80) (Kimura, 1980) evolution model within the software package Geneious (Settings: Transitions/Transversion − Estimated = 4; Proportion of invariable sites − Fixed = 0; Number of substitution rate categories = 1; Gamma distribution parameter − Estimated = 0).

## Results and discussion

Analysis of 16S rRNA gene sequences generated allowed us to examine the community diversity and OTU abundances of the FeMn nodule-associated and sediment mircoorganisms from four sites in SPG. Analyzing the total diversity of the samples (α-diversity), how the community diversity compared between samples (β-diversity), and the overall community composition functioned as a corollary for establishing putative roles and functions of the microbes associated with FeMn nodules.

### Alpha-diversity

α-diversity is a general measure of species diversity (OTU richness) used to contrast different samples/sites in ecological studies. Results for this study returned a total of 1270 OTUs across the 20 samples. Based on the observed OTU richness, it is apparent that the sediment samples collected from 0 to 5 cm near the collected nodules tended to have a higher number of OTUs (Table 2), though not exclusively. Samples collected from different layers within each nodule demonstrated a wide range in the number of observed OTUs, but these numbers do not coincide with specific source layer. The OTU richness of the inward layers (denoted as inner and core) for nodules collected at sites SPG2 and SPG10 are higher, while SPG9 is more even in richness from both outer and inner layers (Table 2). In general, use of Good's Coverage Estimate suggests that our depth of sequencing, and subsequent subsampling, approximately covers 84–98% of the OTU diversity within the samples, with the lowest coverage generated from the sediment samples (Table 2).

**Table 2:** **Sequence effort and diversity measures for each sample**.

**Sampling site**	**Sample name**	**Sample type**	**No. of sequences after processing**	**No. of sequences estimate**	**Good's coverage**	**Observed richness**
SPG2	5B-1	Nodule—Inner	8550	7144	0.895	176.0
SPG2	1A-1	Nodule—Outer	28941	25893	0.912	134.1
SPG2	1B-1	Nodule—Outer	4127	3698	0.925	115.2
SPG2	3	Nodule—Outer	1710	1372	0.938	109.7
SPG9	7	Nodule—Core	1271	928	0.898	192.2
SPG9	6	Nodule—Inner	1493	1296	0.937	128.7
SPG9	1B-1	Nodule—Outer	6749	4895	0.874	210.6
SPG9	2	Nodule—Outer	1773	1464	0.891	181.7
SPG10	7A	Nodule—Inner	23767	18435	0.863	217.5
SPG10	11	Nodule—Inner	1305	1137	0.900	174.8
SPG10	14	Nodule—Inner	4273	3653	0.914	138.9
SPG10	17	Nodule—Outer	1916	1725	0.958	103.9
SPG10	18B	Nodule—Outer	2801	2227	0.907	184.7
SPG10	24	Nodule—Outer	2898	2528	0.915	131.5
SPG10	25	Nodule—Outer	1469	1282	0.976	78.5
SPG10	26	Nodule—Outer	1162	1009	0.920	135.5
SPG2	1-5	Sediment	8658	7254	0.843	252.6
SPG3	1-8	Sediment	9860	8380	0.854	243.3
SPG9	1-5	Sediment	5597	4667	0.858	218.5
SPG10	1-1	Sediment	4064	3423	0.865	216.8

The data suggest that the sediments are more diverse than the nodules. It is generally assumed that increases in diversity are linked to an increased range of potential energy sources [e.g., increased microbial diversity in estuaries and coastal environments compared to the open ocean (Zinger et al., 2012)]. The increased diversity between the nodule and sediment environment may suggest that the surface sediment, despite having low total organic carbon (D'Hondt et al., 2009), is capable of sustaining more microbial diversity than the nodules due to the availability of potential energy sources. Alternatively, the nodule environment introduces a number of cellular stressors due to the presence of increased metal concentrations that may hinder the growth of a more diverse microbial population.

The data also suggest that the outer layers of the nodules are less diverse than the inner layers. The implication may be that the inner layers are more capable of promoting microbial diversity and growth. This may be counterintuitive if it is assumed that the most influential metabolic process were linked to metal oxidation. The outer layers are the site of active nodule growth and the only region directly in contact with the surrounding organic material (OM) of the sediment, thus the major sites of metal oxidation and access to extant OM. The increased diversity in the inner layers may be the result of metabolic activity linked to metal oxide reduction (or some type of cycling between reduction and oxidation of the metal species). Alternatively, microorganisms in the communities of the inner layers may be entombed, such that the increased diversity is an artifact of multiple instances of organisms colonizing the active outer layers and becoming trapped.

### Beta-diversity

β-diversity is a measure used to compare the communities of different samples/sites. Community variation calculations were performed using three different methods that were computed using: [1] only the differences in the OTUs present (Jaccard dissimilarity); [2] using the common OTUs absolute abundance (Bray–Curtis dissimilarity); and, [3] using the relative abundance of OTUs (θ_YC_). In general, the calculations of statistical significance were in agreement for all possible sample groupings (see Supplementary Material, Appendix Table 1), but only the results of the θ_YC_ calculations will be discussed, as this analysis is more robust in demarcating differences between communities (Yue and Clayton, 2005).

**Figure 1:**
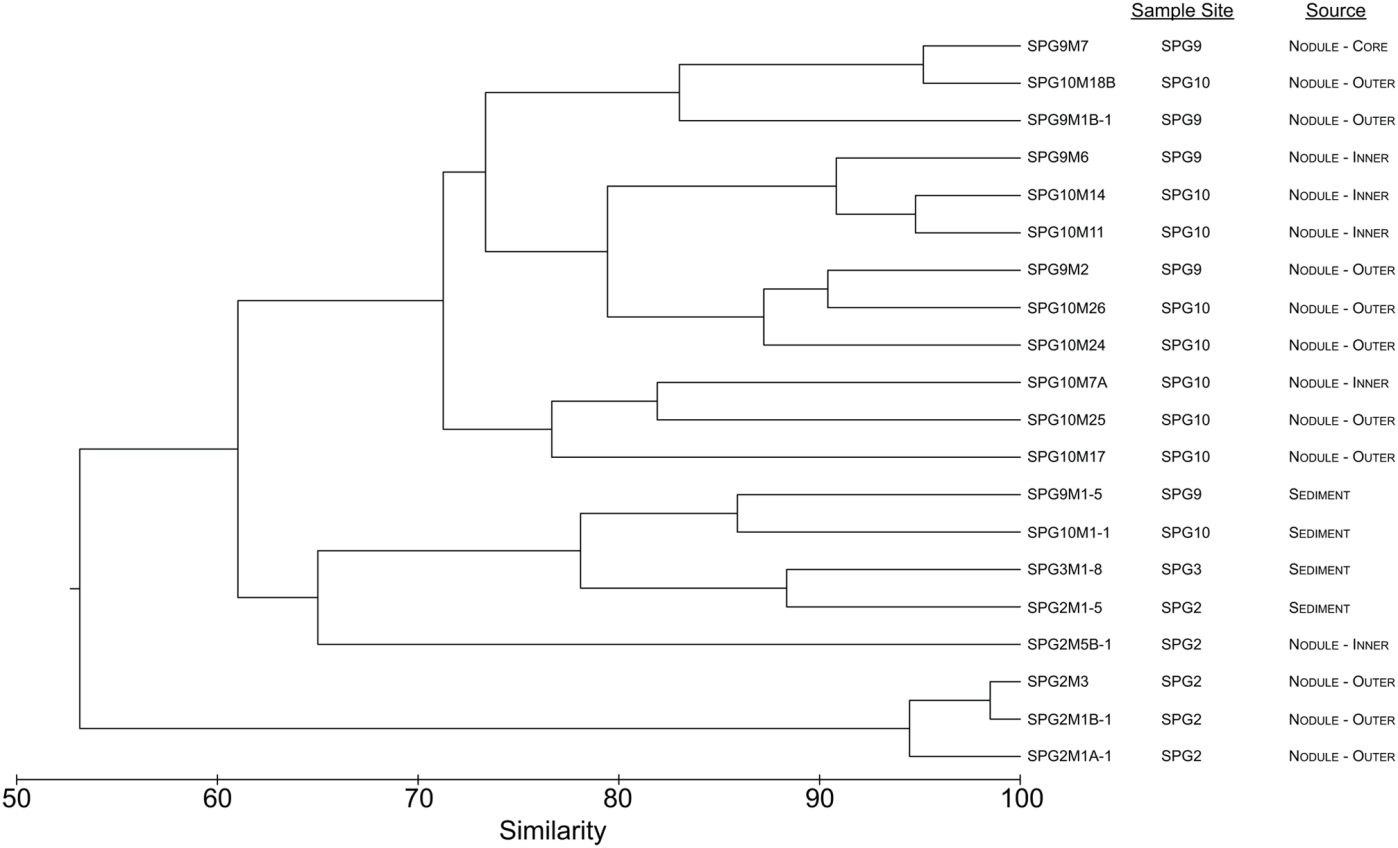
**Dendrogram representing the community similarity of the 20 different sediment and nodule samples using the θ_YC_ calculator in the mothur package.** Samples are classified by sample site and source.

**Figure 2:**
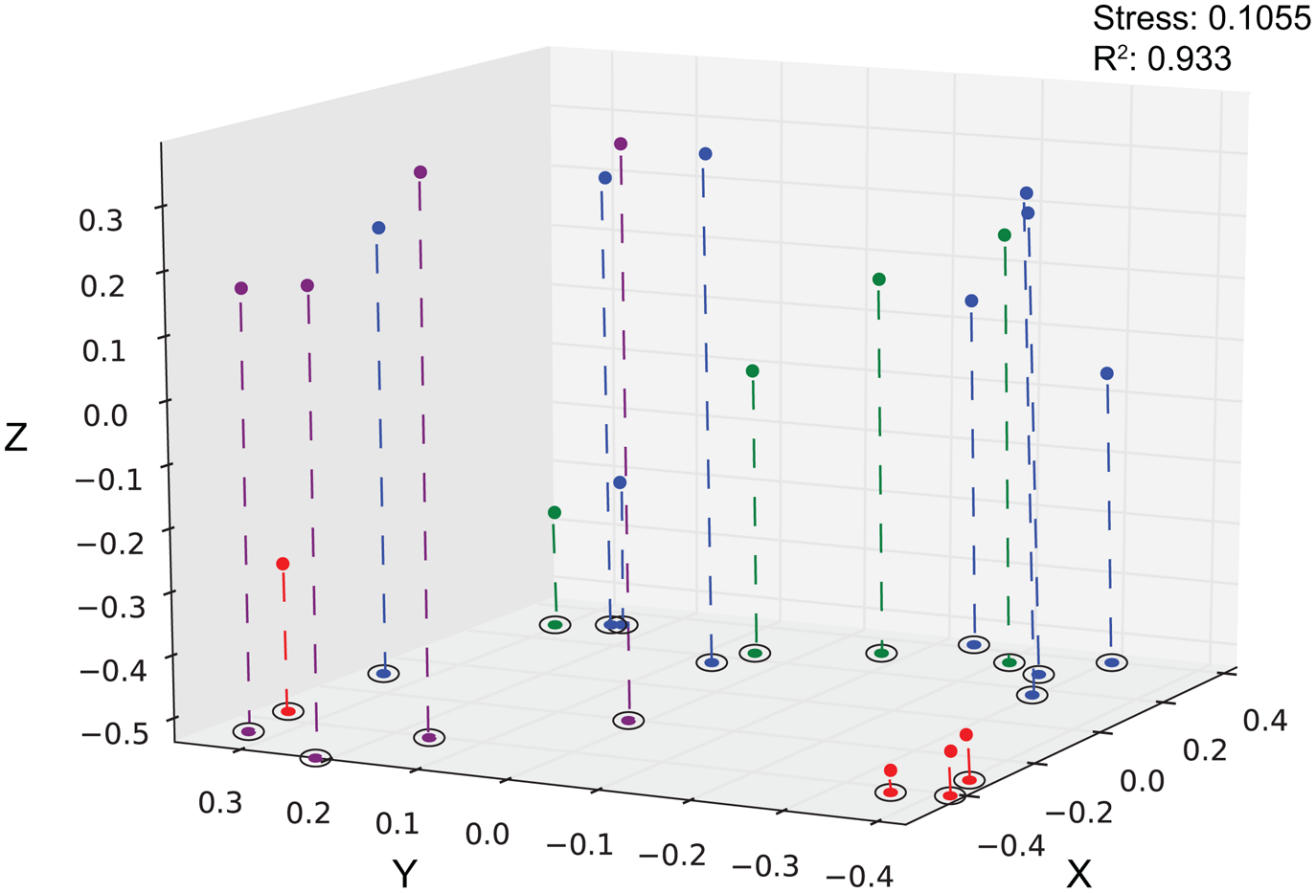
**Three-dimensional plot of the 20 different sediment and nodule samples using the θ_YC_ calculator.** Sediment, purple; SPG2, red; SPG9, green, SPG10, blue. Stress: 0.1055. *R*^2^: 0.933.

Using hierarchical clustering to visualize the differences in communities between the samples and sites, it becomes apparent that SPG9 and SPG10 cluster together and away from SPG2, and that the sediment communities cluster away from the nodule communities, except for an inner layer sample from SPG2 (Figure 1). To increase the resolution of these variations and test the statistical significance, the samples were plotted using NMDS in three dimensions and tested for significance using the AMOVA test (Figure 2). Multiple designations of the samples were used to tease apart which factors attributed the most to community variation. Samples were classified by nodule site or sample source (layer or sediment), individually (e.g., all sediment samples are labeled “sediment” and sample site is ignored), or combined, with various iterations to test significance (see Supplementary Material, Appendix Table 2). Assigning samples by sample site and source allowed for the most accurate interpretation of the data. Many of the broad interpretations made from only labeling by sample site or source were supported for the different iterations of the data.

Each sediment community was significantly different from each of the other sediment communities (Table 3). The sediment communities were also significantly distinct from the nodule communities of SPG2, SPG9, and the SPG10 inner layers, though the SPG10 outer layers were not significantly distinct. This overlap between the SPG10 outer and inner layer communities may be the result of the inaccurate nature of the subsampling process and the difficulty of parsing subsamples that may overlap layers. SPG9 and SPG10 nodule communities were not significantly different from each other, but SPG2 nodule communities were significantly distinct from both SPG9 and SPG10. For both SPG2 and SPG9 the communities on the exterior of the nodule (“outer”) were significantly different from the communities of the inward portions of the respective nodules (for SPG9 this includes an inner layer and a core sample, both distinct from each other and the outer layer). The different layers within SPG10 were not distinct from each other.

**Table 3:** **AMOVA statistical significance results**.

**Sample designation**	**SPG10 inner**	**SPG10 outer**	**SPG10 sediment**	**SPG2 inner**	**SPG2 outer**	**SPG2 sediment**	**SPG3 sediment**	**SPG9 core**	**SPG9 inner**	**SPG9 outer**
SPG10 inner										
SPG10 outer	0.234									
SPG10 sediment	+	0.006								
SPG2 inner	+	0.004	+							
SPG2 outer	+	+	+	+						
SPG2 sediment	+	0.004	+	+	+					
SPG3 sediment	+	0.004	+	+	+	+				
SPG9 core	+	0.003	+	+	+	+	+			
SPG9 inner	0.237	0.661	+	+	+	+	+	+		
SPG9 outer	0.191	0.913	+	+	+	+	+	+	+	
SPG9 sediment	0.232	0.007	+	+	+	+	+	+	+	+

^+^Denotes a statistically significant result less than the Bonferroni pair-wise error rate of 0.0009.

**Figure 3:**
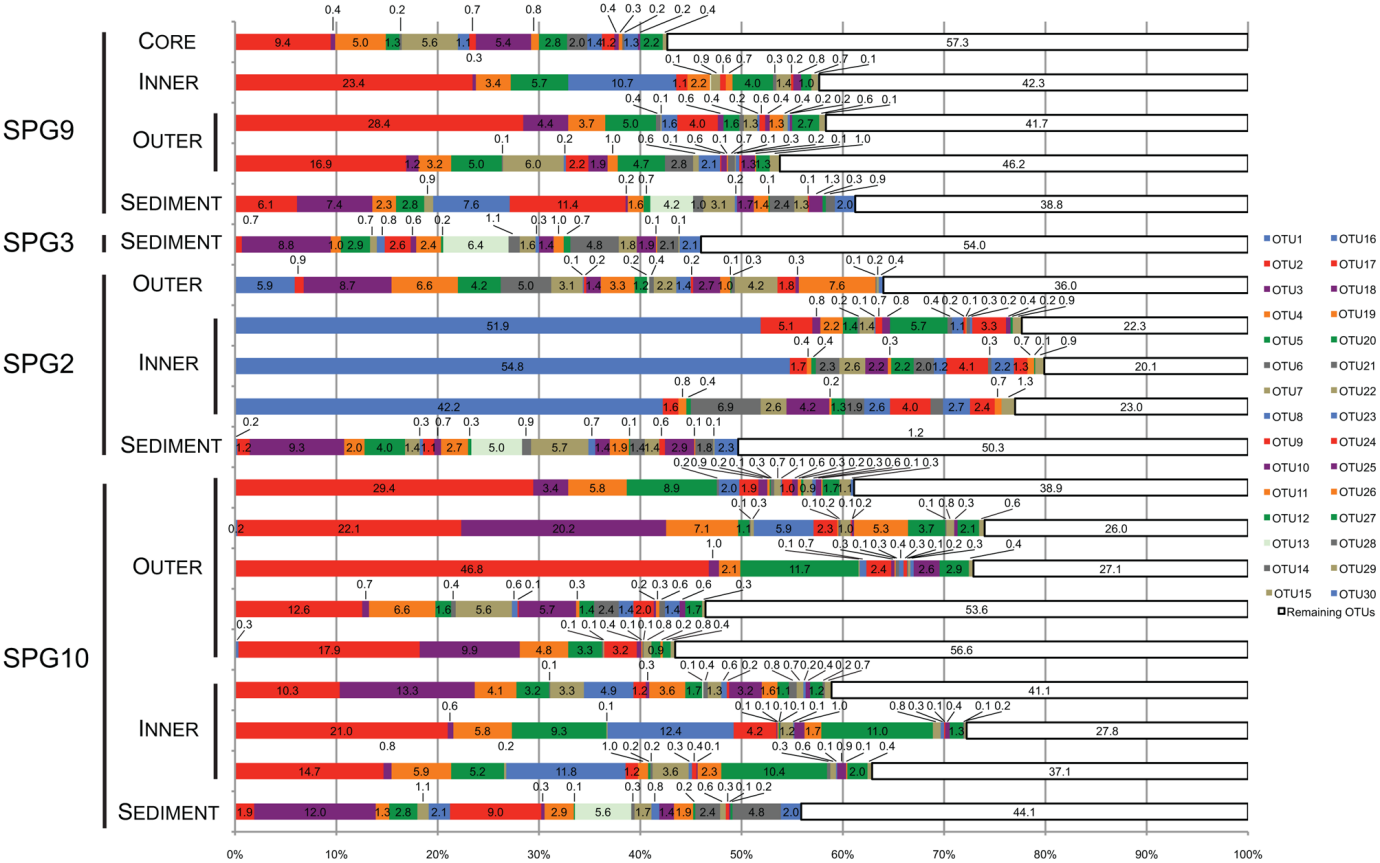
**Stacked bar graph illustrating the percent (%) abundance of the 30 most abundant and the value remaining in all other OTUs for all samples.** Each of the 30 most abundant OTUs was putatively assigned to the lowest taxonomic level possible. Assignments were as follows: OTU2, 3, 4, 5, 8, 9, 13, 15, 19, 20, 22, 25, 27, 28, and 30, *Thaumarchaea* MG-1; OTU7, 14, 16, 18, and 29, *γ-Proteobacteria*: *Sinobacteraceae*; OTU7, 10, 14, 16, 18, and 29, *γ-Proteobacteria: Sinobacteraceae*; OTU6 and 26, *Bacteroidetes*: *Flavobacteriaceae*; OTU17 and 23, *α-Proteobacteria*; OTU1, *γ-Proteobacteria*: *Colwellia*; OTU11, *α-Proteobacteria*: *Rhodospirillaceae*; OTU12, *γ-Proteobacteria*: “endosymbiont”; OTU24, *γ-Proteobacteria*: *Alteromonodales*: NB-1d.

Nodules from SPG9 and SPG10 do not have significantly distinct communities, despite a distance of ~600 km. This is in contrast to the nodule from SPG2, which is significantly different and located at least ~2100 km from SPG9 and SPG10. This difference appears to be related to specific OTUs that are part of the SPG2 nodule community and not part of the SPG9 and SPG10 nodule communities (Figure 3). Sites SPG9 and SPG10 are in a slightly different regime within the SPG, with higher average surface chlorophyll concentrations compared to SPG2 (D'Hondt et al., 2009). Though, if the apparent similarity between the nodules of SPG9 and SPG10 (and difference of nodules from SPG2) were a result of the physical/biological parameters of the overlying water column and OM inputs, it might be assumed that the sediments from SPG9 and SPG10 would have similar communities, when the data indicate these communities are distinct. Possibly the distinct communities are the result of differences in age of the nodules, the surrounding sediment environment, or the seeding populations. There are a number of OTUs that are present in all three of the nodule communities, and it may be that these members play a role in nodule formation/maintenance, while the differences represent possible flexibility in the recruitment of microbial populations.

For the nodules from SPG2 and SPG9 it is possible to differentiate between the inner layers of the nodule and the outer layers. The implication from these results suggests that the interior conditions of the nodule may be selecting for a particular community composition that is different from the community composition of the exterior samples. Many of the community members are present in both the interior and exterior samples, and it is the level of abundance that changes. An increase in abundance may be linked to changes in the activity or role played by these OTUs, such that their metabolisms are favored in the interior conditions compared to other OTUs that decrease in abundance.

### Community composition

The most abundant OTU signatures were examined to determine if the presence/absence of putative taxonomic assignments revealed information about nodule-microbe interactions. For 16S rRNA gene surveys, much of the emphasis is on the most abundant OTUs as a proxy for the most abundant organisms present in the samples. In general, this type of abundance data agrees with the more active members of the community, but there have been examples where the counter is true (Campbell et al., 2011). The top 30 most abundant OTUs (in total sequences assigned to the OTU) were assessed for the role they play in differentiating between different samples and were assigned putative taxonomic groups. Taxonomic assignments were used to predict how the microbial communities might be functioning based on the metabolisms of related organisms. The top 30 OTUs cover 43–80% (mean: 61%) of the total number of sequences assigned to OTUs for each sample (Figure 3). Many of the top 31–50 OTUs contain less <5% of the total abundance, with the rest of OTUs containing <1% of the total abundance for each sample.

Twenty-two of the top 30 most abundant OTUs were present in both the sediment and nodule samples (Figure 3). The sediment samples have three OTUs found only in these samples (OTU13, 28, and 30). Based on the SILVA taxonomy and assignment by mothur, these OTUs were putatively assigned to the MG-1 group within the Phylum *Thaumarchaea* (Figure 3). There were five OTUs not found in the sediment. Three of these OTUs were only associated with SPG2 (OTU1, 6 and 26), while the other two (OTU17 and 23) were present in all of the nodule samples. Of the OTUs only found associated with SPG2, OTU1 could be assigned to the Genus *Colwellia*, and OTU6 and 26 were assigned to the Family *Flavobacteriaceae*. For the other nodule-associated OTUs, both OTU17 and 23 were assigned to the Class *α-Proteobacteria*. Interestingly, 15 of the 30 most abundant OTUs were assigned to the MG-1 *Thaumarchaea* and 6 to the Family *Sinobacteraceae* in the Class *γ-Proteobacteria*.

The OTUs associated only with SPG2 nodule were assigned to phylogenetic groups that are known to specialize in the degradation of high molecular weight compounds (Cottrell and Kirchman, 2000; Methé et al., 2005). While such a function would be common in sediments with higher loads of OM, the SPG sediments are the most carbon-poor marine sediments sampled to date (D'Hondt et al., 2009). Organisms specialized in OM degradation would need to effectively process recalcitrant material. Of all the OTUs, OTU1 was assigned the highest taxonomic rank, putatively a member of the Genus *Colwellia*, and was the most abundant OTU in its respective sample (>50% abundance) (Figure 3). The members of the Genus *Colwellia* are all psychrophiles, while certain members have been shown to form biofilms, possess co-enzyme F420, which may play a role in aromatic compound degradation, and contain the potential for denitrification (Methé et al., 2005). Aromatic compounds are generally more recalcitrant than other compounds, so if OTU1 also possesses such a genetic potential, it may be a viable metabolism for effective growth in the SPG. Interestingly, denitrification is an anaerobic metabolism, but the SPG has been shown to have measurable O_2_ throughout the sediment column (D'Hondt et al., 2009). The other two OTUs unique to SPG2 were assigned to the *Flavobacteriaceae*, known to be a group associated with marine snow particles and key players in the microbial loop of the surface ocean (Cottrell and Kirchman, 2000; Kirchman, 2002; Grossart et al., 2005). Potentially, what makes the SPG2 nodule communities distinct were the members with a role in OM degradation, and not members with putative roles in metal chemistry. This may indicate that the SPG2 community is undergoing different processes biologically, and potentially chemically, than the nodules from the other sites.

Less clearly defined were the OTUs that are exclusive to all of the nodule communities (Figure 3). Both OTU17 and 23 could only be assigned to the level of Class within the *α-Proteobacteria*. The *α-Proteobacteria* is a very large and diverse phylogenetic group. A number of the organisms within the *α-Proteobacteria* partake in metal biogeochemistry, including organisms in the Genus *Magnetospirillum*, which can form magnetosomes composed of magnetite [Fe(II,III) oxide] (Matsunaga et al., 1991). OTU11 was assigned to the Family *Rhodospirillaceae*, for which the Genus *Magnetospirillum* is a member.

Most surprising of the putative taxonomic assignments was the breadth of diversity recovered from the *Thaumarchaea* MG-1 group. Half of the top 30 most abundant OTUs were assigned to this group, including the three OTUs exclusive to the sediment communities. The *Thaumarchaea* are one of the most abundant groups of organisms on the planet and, currently, all members are believed to be capable of the first step of nitrification (ammonia oxidation) and carbon fixation (Hatzenpichler, 2012; Tully et al., 2012). While there is no known direct link between this group of organisms and metal chemistry, their presence in the SPG may have a consequential impact. The SPG sediment environment is believed to be extremely carbon depleted, but with relatively high concentrations of reduced nitrogen compounds (D'Hondt et al., 2009). If the *Thaumarchaea* from this study are active and autotrophic, they may function as a source of reduced carbon, acting as a primary producer for microbial communities.

**Figure 4:**
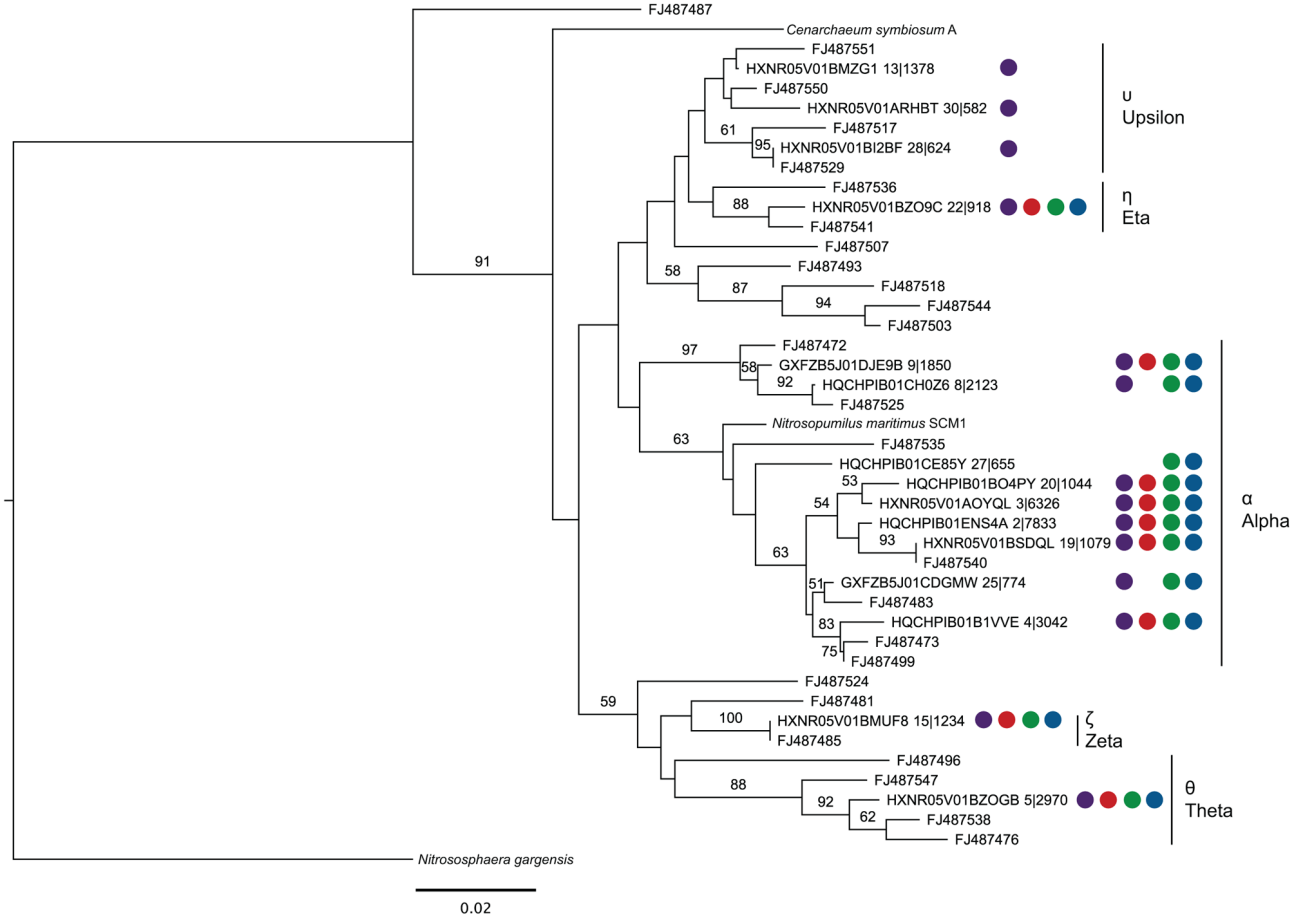
**Maximum likelihood phylogenetic tree constructed using 16S rRNA gene sequences (289 bp) covering the V4 hypervariable region from representatives of each OTU putatively assigned as belonging to the *Thaumarchaea* MG-1 group (Sequence ID OTU No.|No. of total sequences assigned to OTU), sequences from Durbin and Teske (2010) (Accession No.), and sequenced members of the Phylum.** Colored circles correspond to the samples represented by the OTU (at least one subsample must have >0.5% abundance). In fixed order: sediment, purple; SPG2, red; SPG9, green; SPG10, blue. Scale bar: 0.02 changes per site. Bootstraps: 1000.

Using representative sequences for each of the 15 OTUs of the top 30 OTUs assigned to the *Thaumarchaea*, a phylogenetic tree was constructed by including sequences from *Thaumarchaea* genomes and SPG site 12 sediment, where a targeted 16S rRNA gene survey analyzing the *Thaumarchaea* MG-1 diversity was done (Durbin and Teske, 2010) (Figure 4). Based on the phylogenetic groups assigned in Durbin and Teske (2010), OTU13, 30 and 28 fall within the MG-1 Υ group. The MG-1 Υ group was seen to increase with sediment depth and could not be found in the bottom water samples. Most the sequences fell within the MG-1 α group, which were found in the Durbin and Teske (2010) samples at the sediment-water column interface. The phylogenetic data from the nodules and sediment from SPG2, 3, 9, and 10 for the *Thaumarchaea* does not reveal any new distinct groups and covers many of the surface sediment related groups previously identified.

## Conclusion

The results of the study reveal novel information regarding the types of microorganisms associated with FeMn nodules and present a starting point for further research into the role biology plays in their formation and maintenance. It clearly shows that the FeMn nodule-associated microbial community is significantly different from the surrounding sediment communities, suggesting the microbes in the nodules may play different metabolic roles than those of the sediment (and are not just “hitchhikers” from the surrounding environment). This idea is further supported by the underlying similarity between FeMn nodule communities (with some exceptions), especially for nodules from SP9 and SPG10, despite the distance between the sites. Furthermore, the communities associated with the inner portions of a nodule are distinct from its outer portions and surrounding sediment, implicating a possible selective pressure, such that the dominant physiologies of the inner nodule are different than those of the outer nodule. This may be the result of a shift from metal oxidation and OM degradation on the exterior to metal oxide reduction in the interior, or potentially some form of complex cycling of metal species and OM. The presence of sequences related to predominantly OM degrading organisms for the SPG2 nodule suggest an increased role in heterotrophic metabolism for these samples. The presence of *Thaumarchaea* in all samples may highlight a possible source for a food web supported by ammonia oxidation and carbon fixation in the energy-limited SPG environment.

The lack of an abundant 16S rRNA gene sequence strongly linked to a phylogenetic group with known metal metabolisms may have implications for the role biology plays in nodule chemistry. It could be possible that the microorganisms associated with FeMn nodules do not play an active role in nodule formation through key metabolic functions (e.g., metal oxide reduction as a terminal electron acceptor in a electron transport chain), but the biochemical reactions associated with microorganisms may still be important. Oxidation/reduction of the metal species may be ancillary biochemical mechanisms related to biofilm formation/maintenance, cellular detoxification of reduced metals, or metabolic waste sequestration. Alternatively, the nodule-associated organisms may be utilizing the OM degraded by the FeMn nodule to sustain growth and nodule formation is truly abiotic. Further study of the genomic potential of the microbial community may reveal metal metabolisms in unexpected lineages or biological mechanisms linked to nodule chemistry.

### Conflict of interest statement

The authors declare that the research was conducted in the absence of any commercial or financial relationships that could be construed as a potential conflict of interest.
