# Supplementary figures and images for: Microbial communities associated with ferromanganese nodules and the surrounding sediments
Source: Front Microbiol. 2013 Jun 25;4:161. doi: 10.3389/fmicb.2013.00161 (PMC3691505; doi:10.3389/fmicb.2013.00161)

# South Gyre FeMn Nodule Technical Replicates

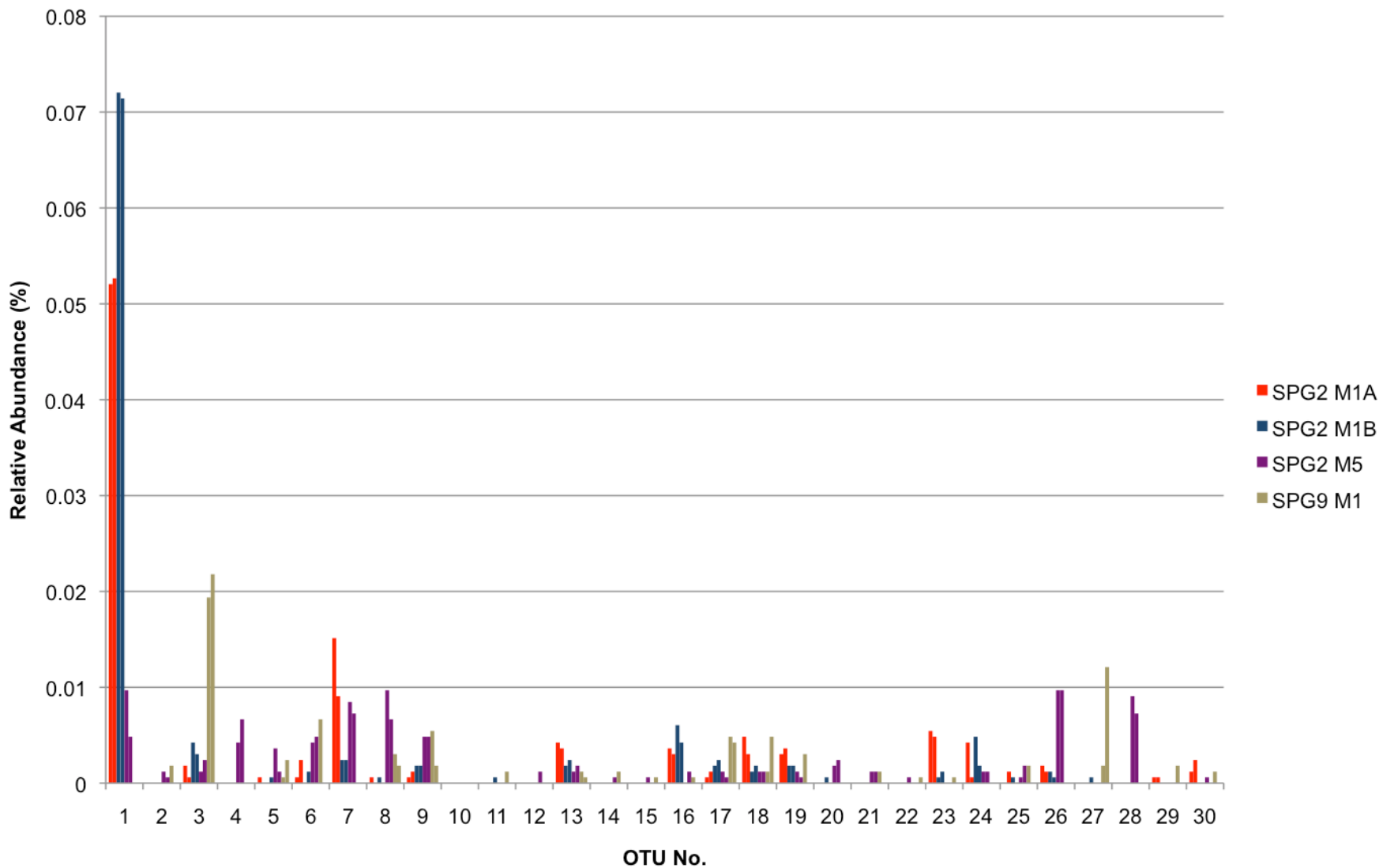

Supplement: Supplementary file 3 [file DataSheet3.PDF]
